# Moral zombies: why algorithms are not moral agents

**DOI:** 10.1007/s00146-021-01189-x

**Published:** 2021-04-16

**Authors:** Carissa Véliz

**Affiliations:** 1 Institute for Ethics in AI, Faculty of Philosophy, Hertford College, University of Oxford, Oxford, UK

**Keywords:** Algorithms, Moral agency, Moral responsibility, Autonomous systems, Zombies, Accountability, Autonomy, Sentience, Consciousness, Reasons-responsiveness

## Abstract

In philosophy of mind, zombies are imaginary creatures that are exact physical duplicates of conscious subjects for whom there is no first-personal experience. Zombies are meant to show that physicalism—the theory that the universe is made up entirely out of physical components—is false. In this paper, I apply the zombie thought experiment to the realm of morality to assess whether moral agency is something independent from sentience. Algorithms, I argue, are a kind of functional moral zombie, such that thinking about the latter can help us better understand and regulate the former. I contend that the main reason why algorithms can be neither autonomous nor accountable is that they lack sentience. Moral zombies and algorithms are incoherent as moral agents because they lack the necessary moral understanding to be morally responsible. To understand what it means to inflict pain on someone, it is necessary to have experiential knowledge of pain. At most, for an algorithm that feels nothing, ‘values’ will be items on a list, possibly prioritised in a certain way according to a number that represents weightiness. But entities that do not feel cannot value, and beings that do not value cannot act for moral reasons.

## 1 Introduction

In philosophy of mind, zombies are imaginary creatures designed to illustrate issues related to physicalism and consciousness. Zombies are exact physical duplicates of conscious subjects for whom there is no first-personal experience. In addition to being physically just like us, molecule for molecule, they are also our functional duplicates. Zombies behave indistinguishably from human beings—they complain about the weather, they cry when they watch sad movies, and they have been known to discuss philosophy conundrums for hours at a time. Unlike human beings, they lack conscious experience. It is nothing there is like to be a zombie—they do not suffer from cold when it is cold, they do not feel sadness when they cry, and philosophical puzzles do not worry or excite them.

Zombies are meant to show that physicalism—the theory that the universe is made up entirely out of physical components—is false. If there could be creatures that act like us without conscious experience, then consciousness must be something above and beyond our physical bodies.

The zombie thought experiment is yet to be applied to the realm of morality, but there is an analogous question to be explored regarding sentience and moral agency. Applying the zombie thought experiment to moral agency can help us assess whether moral agency is something independent of conscious experience, or sentience. By sentience I mean the capacity, at a minimum, to have a subjective experience of pleasure and pain. I wish to remain agnostic as to whether subjective experience is synonymous with sentience. For the purposes of this paper, I will focus on sentience because it is conceivable that there could be conscious experience without feelings of pain or pleasure, and without emotions, and I take these latter experiences to be what matter most for moral agency. Delving into these issues, however, is beyond the scope of this paper and unnecessary for my purposes.

In what follows, I first present the case for algorithms being akin to moral zombies, and for the significance of the moral zombie thought experiment. I then suggest that much of the literature on algorithms assumes a degree of moral agency in algorithms. In turn, most accounts of moral agency defend either some version of autonomy or some version of moral responsibility as constitutive of moral agency, or both. I go on to analyse whether algorithms and moral zombies are autonomous in the relevant sense for moral agency, and argue that they are not. I then argue that moral responsibility is more relevant to assessing moral agency than autonomy, but neither algorithms nor moral zombies are morally responsible either. I contend that the main reason why algorithms can be neither autonomous nor accountable is that they lack sentience. Moral zombies are incoherent as moral agents because they lack the necessary moral understanding to be morally responsible. To understand what it means to inflict pain on someone, it is necessary to have experiential knowledge of pain. I end the paper by responding to three possible objections.

## 2 Algorithms as moral zombies

Moral zombies would be creatures who act indistinguishably from us as moral agents, but for whom there is nothing it is like to be them. Moral zombies would act like us without feeling like us. Like human beings, they would be able to do good by donating to charity, protecting vulnerable people against injustice, and respecting rights. They would also have the power to do evil by violating rights and causing unnecessary suffering—they could insult, betray, threaten, and physically aggress others. Unlike human beings, they would not feel pain, pleasure, empathy, intimacy, remorse, guilt, shame, or any other moral emotion. A moral zombie would not rejoice at saving a life or suffer guilt from taking one.

Moral zombies, as described, seem to be conceivable creatures, which shows that the power to have a moral impact in the world is independent from sentience. But we already know that from hurricanes and other natural phenomena that can cause harm without there being any agency. The relevant question for ethics is whether moral zombies are conceivable *as moral agents*. If they are not, then the thought experiment would suggest that where there is moral agency, there is sentience.

Moral zombies may not only be a thought experiment. It could be argued that something like moral zombies are already among us; they are often called algorithms, robots, or AI (I will use these terms more or less interchangeably). Automated systems may not look like us (yet), but this detail is arguably morally irrelevant (at least for the question of moral agency). We can imagine robots looking like human beings in the not too distant future. While AI is not yet and will likely never be a physical duplicate of human beings, it can be considered a functional duplicate in some respects—which is why machines can replace human beings in increasingly more tasks. Algorithms already resemble us in some of the decisions they are making, and the moral impact they can have on the world, with people getting hired, sacked, rewarded, and even jailed on account of them. They are not like us, however, in that there is nothing it is like to be them. Thus, thinking about algorithms can help us think through the moral zombie thought experiment, and thinking about moral zombies can help us better understand algorithms.

The first objection someone might level at my argument is that we cannot know for sure that algorithms or machines are not sentient. Admittedly, I cannot prove beyond all shadow of a doubt that there is nothing it is like to be an algorithm. But, for that matter, I cannot prove that rocks are not sentient either. If panpsychists are right, even rocks may have some degree of consciousness or proto-consciousness. There is, however, not enough evidence to suggest that algorithms (or rocks) have a mind or can feel.

It might be argued that, out of epistemic humility, if algorithms behave as us, we should treat them much like we treat human beings (Danaher 2020; Sparrow 2004). At the moment, however, algorithms resemble us only in some of the tasks and functions they perform in society, but not in a more global sense. In that regard, they are not full zombies, but merely something like functional moral zombies. Algorithms today can select job candidates or assess the probability of someone committing a crime, but they cannot develop a passion for French comedies or be moved by kindness.

There may come a time when machines are so similar to us that caution may be warranted (Véliz 2016). It is likely that they would need to have bodies similar to ours. There might not be such a thing as a disembodied mind (Varela et al. 1991). And it might be that only beings with biological bodies can be sentient. Part of what makes us feel and think the way we do is our heart beating faster when we are excited, our blood pressure dropping when we experience sadness, sweat running down our temples when we are scared, our skin curling into goose bumps when we are awestruck. The burden of proof, then, seems to be on whoever wants to argue that algorithms are sentient.

## 3 Moral agency and algorithms

There is a tendency in the literature to describe automated systems in ways that seem to imply some degree of moral agency^1^ (Sharkey 2017). In some cases, implicit references to moral agency seem to be a mere rhetorical device. In the spirit of Daniel Dennett’s intentional stance, sometimes it can be useful to describe artefacts *as if* they had intentions and beliefs, even when we think them mindless. Wendell Wallach and Colin Allen write, for example, that ‘Driverless systems put machines in the position of making split-second decisions that could have life or death implications’ (2009: 14). In the same vein, Robert Sparrow writes that what makes weapons systems automated is ‘that they have the capacity to choose their own targets’ (2007: 70). Even though Sparrow goes on to argue that autonomous weapons systems are not morally responsible, the language of moral decision-making seems appropriate only in reference to moral agents. Referring to autonomous systems in ways that are accurate only when describing moral agents is morally significant. As Noel Sharkey puts it:

‘[Some terms] act as linguistic Trojan horses that smuggle in a rich interconnected web of human concepts that are not part of a computer system or how it operates. Once the reader has accepted a seemingly innocent Trojan term (…) it opens the gate to other meanings associated with the natural language use of the term’ (Sharkey 2012: 793).

Perhaps, this tendency to talk of autonomous systems as if they were moral agents, or the messy debate on moral agency and AI, has led some writers to suggest that we should stop asking ourselves if autonomous systems are *truly* moral agents (Behdadi and Munthe 2020).^2^ But that approach seems unsatisfying. The reason we ask ourselves if AIs are or can be moral agents is not out of metaphysical curiosity, but rather because we care about the practical implications. If AIs are not moral agents, then someone needs to take responsibility for what they do. If AIs are moral agents, then presumably we should be treating them accordingly (suing them when they break the law, sending them to jail, and considering them bearers of rights, among other practices).

Although most writers only use the language of moral agency metaphorically or pragmatically, in some cases, there does seem to be an explicit belief that autonomous agents can be moral agents. For instance, when writing about self-driving cars, Mark Coeckelbergh argues that ‘all agency is entirely transferred to the [car]’ (2016: 754).

Of course, different authors mean different things when referring to moral agency. A dominant way of categorising moral agency in the AI literature is that of James H. Moor (Moor 2009). He proposes four categories of ethical agency: (1) *ethical impact agents* (agents whose actions have ethical consequences; most or all autonomous systems count as ethical impact agents); (2) *implicit ethical agents* (those that have been designed with ethics in mind, e.g. ATMs); (3) *explicit ethical agents* (agents that can act *from* ethics, and not merely in accordance with ethics; they can identify ethical problems and work out solutions for themselves); (4) *full ethical agents* (they can do what explicit ethical agents do and, in addition, they have consciousness, intentionality, and free will). My claim in this paper is that explicit ethical agents and full ethical agents are one and the same category. In other words, I claim we will not get an agent that can identify ethical problems and respond adequately to them without sentience.

Given that my argument pivots on sentience, the most interesting perspective (and contrary to mine) is that of Luciano Floridi and J.W. Sanders, who not only argue that artificial autonomous agents can be considered moral agents, but also that they constitute an instance of ‘mind-less morality’ (2004, 351). In disagreement with Floridi and Sanders, I contend that sentience is necessary for moral agency, because conscious experience of the kind that allows for feelings like pleasure, pain, and empathy seems necessary for moral agency.

An agent is, roughly, an entity that can be the source of action. Most accounts of *moral* agency in philosophy defend either some version of autonomy or some version of moral responsibility as constitutive of moral agency, or both. The relationship between these concepts is fraught with controversy. Some philosophers think that whenever there is one, the other follows, while others argue that autonomy and moral responsibility are independent from each other. For our purposes, it is not essential to establish the exact relationship between autonomy and moral responsibility. What matters is that, under any plausible account of autonomy that is relevant to morality, and under any plausible account of moral responsibility, neither moral zombies nor algorithms can be moral agents, or so I argue.

## 4 Autonomy and moral agency

Nomy Arpaly argues that there are at least eight ways in which ‘autonomy’ has been conceptualised in the literature: as personal efficacy, psychological independence, having a moral right to self-determination, authenticity, having a coherent self-image, being heroic, self-governance, and being responsive to reasons. In what follows I go through each of these and argue that many of these accounts of autonomy do not seem relevant to morality, and the ones that do (self-governance and reasons-responsiveness), do not apply to moral zombies or algorithms.

### 4.1 Personal efficacy

This sense of autonomy describes the quality of not depending on other people’s help to navigate the world. Floridi and Sanders contend that algorithms are autonomous because they are ‘able to change state without direct response to interaction’—that is, they can act independently of the human beings who created them (2004: 357). It does not seem that this sense of autonomy is at all relevant to assess whether algorithms are moral agents, however. Tornadoes can also go about their business without human help, and that tells us nothing about whether they are agents—much less moral agents.

Autonomy in moral and political philosophy is a much richer concept than in the context of computer science and engineering. It is the richer kind of autonomy that is relevant for moral agency, and not to be confused with the looser sense of the adjective ‘autonomous’ used when talking about technology. Once we identify an entity as a moral agent, or as the subject of moral rights, then it might be relevant to know what they can and cannot do independently of other agents (e.g. to assess how responsible they can be for their actions, or to investigate whether they are owed assistance in virtue of their limitations, etc.). But we are not there yet when it comes to moral zombies or algorithms, and knowing whether they are personally efficacious tells us nothing about whether they are moral agents.

### 4.2 Independence of mind

This version of autonomy is similar to the previous, except instead of focusing on the ability to *act* independently of others, it is concerned with the ability to *think* independent of others. If by ‘mind’ we mean something like subjective experience, then neither moral zombies nor algorithms have a mind. Some philosophers, however, might want to argue that subjective experience is not necessary to have a mind. In any case, it seems that mental independence does not help as a criterion to establish moral agency. Computers that play chess, for instance, can ‘think’ independently of human beings, and it is quite clear that they are not good candidates for moral agency. Once it is evident that someone is a moral agent, it may then be morally relevant to establish whether they have been unduly influenced in a particular circumstance (through propaganda, or subliminal messages, or direct brain stimulation). But, again, independence of mind is not relevant as a test of moral agency.

### 4.3 Moral right to self-determination

It is widely accepted that moral agents have a right to self-determination. As a competent adult, you should be able to decide how you live your life—as long as you do not violate other people’s rights. Respect for autonomy is one of the pillars of bioethics. It is what allows patients to decide which treatment, if any, they are to receive. While the right to self-determination is a very important one, it assumes moral agency on the part of the right bearer. The right to self-determination is a tool to protect moral agents, it is not a property of agents, and as such it cannot be a criterion for moral agency.

### 4.4 Authenticity

To what extent someone acts in an authentic way—following their true convictions, or being true to who they are—as opposed to acting impulsively, seems to matter for morality. Harry Frankfurt (1988) argued that some cases that could be interpreted as cases of weakness of will are actually cases of someone being constrained by their own deepest values. In response, David Velleman (2002) has argued that such a theory is not about autonomy but about authenticity. Whether authenticity is part of autonomy is not important for our purposes. While authenticity might be important to assess character and the moral significance of actions, it does not seem like a good test for moral agency. We can imagine inauthentic moral agents that are moral agents nonetheless. Consider a person who does not trust her deepest convictions and instead acts imitating others. Such behaviour makes her less admirable as a moral agent, but her inauthenticity, unless it were caused by some cognitive deficiency that would question her competence, is no reason to question her moral agency.

### 4.5 Self-identification

Given that autonomy is often thought of as related to the absence of external pressure, the phenomenological experience of owning one’s desires and acts is often thought to be relevant to autonomy as well. The autonomous person, on this account, is one who is able to self-identify with her desires and has a coherent self-image. Most people have done something at some point in their lives that was out of character for them, with which they did not self-identify. While the degree of self-identification can be relevant to judge the moral significance of an action, it does not seem relevant to judge whether someone is a moral agent. Suppose that, even though you are usually a very calm person, one day you lose your temper and scream at your partner. While you might not self-identify with that action, that does not strip you of your moral agency. Self-identification, then, is again not an appropriate test of moral agency.^3^

### 4.6 Heroism

Many accounts of autonomy have an ideal agent in mind. For stoics, the ideal agent is one who exercises ataraxia; for Aristotle, it is the person who leads a life of contemplation; for Nietzsche, it is the free spirit. Having an ideal can help us assess how close or far a person or an action is from the best it could be, but it cannot be a criterion for moral agency. If only the few who reach the ideal were to be considered moral agents, most competent adults would fail the test of moral agency.

### 4.7 Self-governance and reasons-responsiveness

One of the most popular ways of thinking about autonomy is in terms of self-governance. The term ‘autonomy’ is derived from the Greek ‘autos’ (self), and ‘nomos’ (law); as such, the concept that the term ‘autonomy’ aims to capture seems to be, broadly speaking, the property of self-government, characterised by the agent’s ability to decide how to act. Even within theories of autonomy as self-governance, there is a great deal of controversy and variety.

The idea of autonomy as self-governance can be traced back to Immanuel Kant (2019), for whom autonomy means that no authority external to ourselves is needed to dictate the demands of morality (Schneewind 1992). Being autonomous entails imposing on ourselves the demands of morality: we have the ability to recognise what is the right thing to do and to act accordingly.

I discuss autonomy as self-governance and as reasons-responsiveness together because they seem intimately related. More precisely, it seems that self-governance requires reasons-responsiveness. Moral agents make decisions and act accordingly at least partly because they are responding to reasons. If an entity is not the kind of creature who can understand reasons, then she will not govern herself in a way that is relevant for morality.

Crucial to autonomy as self-governance is the capacity to act in accordance with reason in a way that responds to one’s own motives (Christman 2015). To be autonomous, one must be able to reflect on (Watson 2013: 4–5), endorse, and act on one’s values (Christman 2015). It is because a person is able to choose her values for herself and live accordingly that we must ask for her consent to interact with her in invasive ways, for example, in the case of a medical procedure.

We do not need to ask an algorithm its permission to modify it or even terminate it because algorithms do not have values of their own; they do not care about their own existence. Nor can they respond to reasons qua reasons: You cannot *persuade* an algorithm to do something through giving it good reasons—you can only programme it one way or another.

A robot could respond to its environment in ways that are in accordance with ethics (e.g. if it sees a human being carrying something heavy, it offers to carry it for them). That a human being is struggling with carrying her groceries and that it will be effortless for the robot to carry them for her, however, is not a *reason* for the robot—it is an instruction. The robot cannot *desire* to relieve the strained arms of the person because, first, it has no desires, and second, it has no idea what it feels like to have your arms hurt from carrying something heavy. Furthermore, the robot cannot reflect on the relationship between our acts and having a healthy citizenry, or on the benefits of civil friendship. A reason, for moral agents, amounts to something that *matters* to us, that we *care* about because we understand its moral significance.

Algorithms are programmed to do something: win a game of chess, distinguish spam from non-spam, identify people who might want to buy a product, assess whether a candidate will be appropriate for a job, etc. Algorithms, however, are incapable of normatively assessing the objective for which they have been created, and modifying their behaviour accordingly.^4^

Consider the role algorithms play in advancing for-profit colleges in the United States. These are expensive, low-quality colleges that advertise themselves to vulnerable populations as a way out of their underprivileged status. In fact, in the work market, a person is no better off having a diploma from a for-profit college than not having attended college at all (Darolia et al. 2015). To identify possible clients for a for-profit college, algorithms look for people in the poorest postal codes who have clicked on ads for payday loans, or whose search histories reveal a concern with post-traumatic stress (O’Neil 2016: Loc 1052). When such algorithms perform their tasks, they are not wondering whether it is morally correct to prey on vulnerable people, and they are incapable of deciding to quit their jobs and go for a more ethical line of work.

A self-driving car is incapable of choosing its destination on a whim. It cannot wake up one day with the desire to enjoy the countryside and disobey its owner, who needs to get to work. A killer robot cannot become a pacifist after considering the negative consequences of its actions. It is not that the killer robot has been programmed to believe that its killing is morally justified—it does not have the capacity to either believe in or question its *raison d’être*. It cannot reflect on what it wants, what is worth pursuing, or how it should live its life.^5^

In a nutshell then, algorithms are neither self-governing, because they need external input to set themselves goals, nor reasons-responsive, as no reasons could ever ‘convince’ them to change the goal for which they have been programmed.

Moral zombies, on the other hand, might *appear* to be both self-governing and reasons-responsive, given that, by definition, they would behave indistinguishably from human beings. However, it is unclear that moral zombies could be said to have motives of their own. If nothing *moves* them, if they cannot feel desire, fear, hope, or empathy, it could be argued that they cannot own their goals like moral agents do. In that sense, whatever goal they pursue is not theirs, in that they do not have the capacity to endorse it, to feel they approve of it. Similarly, it is questionable that moral zombies are reasons-responsive, at least in some moral cases. Suppose a human being asks a moral zombie to stop stepping on her foot because it is painful to her. If the moral zombie has never felt pain, it is unclear that we should say that when it stops stepping on the person’s foot, it is responding to the reasons given by her.

According to our analysis thus far, only self-governance and reasons-responsiveness are relevant to the kind of autonomy that in turn is suggestive of moral agency, and it seems like neither moral zombies nor algorithms are autonomous is those senses. Focusing on autonomy, however, although intuitive, may not the best way of getting at whether moral zombies or algorithms are moral agents. First, there are so many senses of autonomy, some of which are hard to separate from each other, that a focus on autonomy in moral discussions risks inviting misunderstanding, rather than contributing clarity (Arpaly 2002, 126). Second, working out who is a moral agent is not primarily a matter of intellectual curiosity—it is a practically-oriented task. What we ought to look for then, if we want to know whether there can be moral agency without sentience, is a satisfactory account of moral agency.

There are two main practical reasons why we might care about ascertaining moral agency. The first reason is to protect subjects of moral rights who might not be able to protect themselves. In medical ethics, for instance, we want to make sure that research subjects and patients are in a position to make competent and well-informed decisions that they are unlikely to regret in the future. In that context, establishing autonomy is a priority because autonomy is a sign that such people can decide for themselves what is best for them, and therefore should be allowed to make such decisions. That worry does not apply to either moral zombies or algorithms. Given that moral zombies and algorithms do not have the capacity to suffer, we do not worry about them regretting their decisions, or unwittingly bringing harm to themselves.

What is concerning about algorithms is that they can act on the world and have enormous impact, for better or worse, which leads us to the second reason why ascertaining moral agency is important. When things go wrong, we want to make sure that we know who to look to when wanting to secure accountability. Given that establishing who is a moral agent is a practical challenge, I will stay clear of metaphysical issues (for example, regarding free will). I take it that our moral and political practices regarding moral agency are grounded enough that metaphysical questions will not be relevant for practical purposes. Establishing moral responsibility, then, is important in contexts in which we want to make sure the appropriate parties are held accountable for possible wrongdoings.

In other words, philosophical discussions of moral agency are a coin with two sides: one side has the decision maker as the object of concern (autonomy) and the other side is concerned with accountability (moral responsibility). In the context of both moral zombies and algorithms, what matters is accountability.

## 5 Moral responsibility and moral agency

Most accounts of autonomy are not relevant to establish moral responsibility. Agents can be morally responsible even if they do not have much independence of mind or body, or when their moral right to self-determination has been violated (e.g. slaves can be morally responsible agents), even if they are not moral heroes, even if they do not always identify with their action, and even if they are inauthentic. In contrast, both self-governance and reasons-responsiveness seem to be important for moral agency, but not necessarily because they are important for autonomy—unless we take autonomy and moral agency to be synonymous, which would go against the term usage of most philosophers. Rather, self-governance and reasons-responsiveness are important for moral agency insofar they are capacities that ground moral responsibility.

Gary Watson (2013) convincingly argues that moral agents are beings who are autonomous (in the sense of self-governing), and accountable (morally responsible in a way that they are answerable to others). Happily, there is much more consensus about what moral responsibility is than about autonomy.

Michael McKenna understands morally responsible agency as ‘accountability for guiding conduct in accord with the demands of morality’ (2013: 206). Accountability is intimately related to notions of moral blameworthiness and praiseworthiness. According to Arpaly, ‘any agent who is morally praiseworthy or blameworthy for her action is, by definition, at least somewhat morally responsible for that action’ (2002, 129).

Just as algorithms are not autonomous, they are not accountable. As accountable beings, ‘we are answerable to others for how we lead our lives’ (Watson 2013: 1). That is, we can recognise others’ interests and moral claims, and when we do not respect them, we are liable to be the subjects of blame or even punishment. An algorithm, in contrast, does not think about the suffering it might be causing by encouraging vulnerable people to take out heavy loans to pay for a degree at a for-profit college that is worth little or nothing. When wronged by an algorithm, it would not occur to us to punish it or ask it for compensation. Rather, we would seek redress from the people who designed, implemented, and were supposed to supervise the algorithm.

Floridi and Sanders argue that we should not confuse accountability with responsibility. According to them, ‘[a]n agent is morally accountable for *x* if the agent is the source of *x,*’ where *x* is an action causing moral good or evil. To also be morally responsible, they argue, ‘the agent needs to show the right intentional states’ (Floridi and Sanders 2004: 371). They believe that entwining the concepts of accountability and responsibility amounts to ‘confusing the *identification* of *x* as a moral agent with the *evaluation* of *x* as a morally responsible agent’ (367). Morality is intrinsically about normative evaluation, however. If a moral agent can be identified as such, then it must also be the case that we can evaluate her as responsible for her actions.

Moral responsibility is intrinsically tied into moral responsiveness. Arply argues that ‘For an agent to be morally praiseworthy for doing the right thing is for her to have done the right thing for the relevant moral reasons—that is, the reasons for which she acts are identical to the reasons for which the action is right’ (Arpaly 2002: 72). When a morally valuable consequence comes about as a result of a lucky accident (i.e. the sun shining), no one is praiseworthy for it.

To identify a moral agent as the source of action is tantamount to her being the appropriate target of praise or blame. It makes no sense to identify someone as a moral agent without evaluating her a responsible moral agent. To be a moral agent just means that one is responsible for one’s moral actions. When moral agents hurt others, we can blame them for their bad intentions or their neglect. In contrast, we do not feel moral outrage against algorithms because they could not have acted otherwise, given their design and input, and they do not have intentions—they do not feel ill will or contempt. ‘If good will—the motive(s) from which praiseworthy actions stem—is responsiveness to moral reasons, deficiency in good will is insufficient responsiveness to moral reasons, obliviousness or indifference to morally relevant factors, and ill will is responsiveness to sinister reasons—reasons for which it is never moral to act, reasons that, in their essence, conflict with morality’ (Arpaly 2002: 79). Unlike people, moral zombies and algorithms cannot act from good or ill will—they are not sentient.

## 6 Sentience and moral agency

I contend that the main reason why algorithms can be neither autonomous nor accountable is that they lack sentience. To have a conception of the good that we want to pursue (autonomy), we need to have a feel for what leads to pleasure, meaningfulness, and satisfaction. To guide our actions by the recognition of others’ moral claims in a way that can count as moral actions (accountability), we must have a sense of others’ capacity to suffer, of what it feels like to be harmed, of what we can do to others’ minds and bodies through our actions.

We do not need to experience every kind of pain to be able to empathise with others’ pain. Someone who has never experienced childbirth can empathise with and desire to alleviate the pain of a woman suffering childbirth pain.^6^ Of course, the closer your experience is to someone else’s, the less of an empathy gap there is likely to be. It is not for nothing that people who are undergoing difficulties can feel special comfort from others who have gone through something similar. But is enough to have a sense of what pleasure and pain are like to act like competent moral agents.

Sentience serves as the foundation for an internal moral lab that guides us in action. When we think about doing something, we imagine the possible consequences we might cause, and consider the kind of pleasure or pain we might create, which motivates us to act one way or another. When we realise we might cause someone great bodily damage, we might flinch as we remember what it feels like to feel physical pain, our stomachs contracting at the thought. When we imagine making someone we love happy, we smile and delight at the prospect partly because we know how pleasant it feels to be happy.

Moral zombies would not act out of the desire to hurt or benefit. What we think of as values will never be values for an AI as long as it cannot feel the warmth of the sun or the sharpness of a knife blade, the comfort of friendship and the unpleasantness of enmity. At most, for an AI that feels nothing, ‘values’ will be items on a list, possibly prioritised in a certain way according to a number that represents weightiness. But entities that do not feel cannot value, and beings that do not value cannot act for moral reasons. Moral zombies, therefore, are incoherent. Zombies might act in ways that harm and benefit human beings, but they could never be moral agents or morally responsible.

My take on moral agency is a Humean one. According to Hume, beliefs on their own are not enough to morally motivate us into action. We need sentiments, passions, to be motivated to act morally (T 2.3.3.4/415, T 3.1.1). If algorithms do not have access to subjective experiences that are attached to value, then they will lack moral motivation because they will be incapable of appreciating moral reasons.

Many accounts of moral agency and responsibility seem to implicitly support the view that sentience is a requirement for moral agency and responsibility. For example, Harry Frankfurt argues that a free agent is ‘prepared to endorse or repudiate the motives from which he acts … to guide his conduct in accordance with what he really cares about’ (1999: 113). Similarly, David Shoemaker notes that ‘the emotions we have make us the agents we are’; ‘without the ability to feel, one would (by definition) be without the capacity to care, leaving one’s decision-making landscape flat and without salience. With no emotional investment in what one might do, all options are on an equal footing’ (Shoemaker 2003: 94, 114). According to Arpy, for an agent to be morally responsible, she must care about morally relevant considerations, and morally relevant features of situations must be able to motive the agent into action: ‘One cannot blame or praise a creature who cannot be expected to perceive the morally relevant features of situations any more than an elephant can be expected to perceive legal factors [or] aesthetic factors’ (2002, 131).

One reason why sentience has not gained more prominence in the literature on moral responsibility might be that, up until now, barring natural causes like old age and the weather, only other human beings could be the cause of phenomena like harm and injustice. It was not as important to focus on sentience because it was a given. Only human beings made decisions about our lives that could negatively affect us, and human beings are evidently both sentient and moral agents. Moral zombies were just a theoretical possibility. Now that algorithms make up a significant source of society’s decisions, we have more of a reason to think about the role of sentience in moral agency and responsibility.

## 7 Responding to objections

### 7.1 The functional equivalence objection

A functionalist about morality would argue that there is nothing more to being a moral agent than acting like one. Such a critic might argue that all we need for algorithms to be moral agents is to have them behave as such, to be functionally equivalent to us. If they *seem* to respond appropriately to moral reasons, make decisions that minimise harm, and are able to modify their behaviour in response to criticism and punishment, then they are moral agents.

In the literature on intelligence, the critic goes on, the worry that there could not be intelligence without subjective experience has lost its force with the passage of time. John Searle (1980) designed his Chinese Room thought experiment to argue that, while computers may be programmed in a way that mimics understanding, computational rules do not produce real understanding. Although Searle’s thought experiment first inspired an explosion of literature around it, interest has tapered. We seem to have stopped caring about whether digital assistants and computers really understand—it is enough for us that they do as we tell them. We seem to be more and more comfortable talking about intelligence without sentience. Why is functional equivalence not enough for moral agency?

My first concern with this objection pertains to non-ideal theory: we might never succeed in designing algorithms that are full functional equivalents to human moral agents. The moral zombie thought experiment might always remain a thought experiment. Even if algorithms can replace human beings in decision-making tasks, it is doubtful that they will ever be able to replicate human moral judgment. My take on sentience supports the view that morality is not codifiable. According to the codifiability thesis, ethics could be summed up in a set of moral rules that could be applied by anyone, independent of their moral competency. It’s unusual, however, to think that someone can be moral as long as they follow a rule, even if they lack understanding or wisdom. Morality seems more of a know how than a know that.

Algorithms mimicking moral agency will respond to a set of instructions, and sets of instructions can, at best, be proxies for moral reasons. The worry is that proxies might not be good enough. A computer scientist could programme an algorithm to behave in such a way as to not make people frown or cry (a proxy for not making people suffer), thereby roughly behaving in a morally acceptable way. Except, sometimes, making another cry is precisely the moral thing to do, as when we vaccinate children.

One could of course imagine inserting exceptions into the algorithm—e.g., do not make people cry unless they are children and need to be vaccinated—but the algorithm will still be pursuing proxies and not morality itself. While it might get it right sometimes or even most of the times, other times, when it has not encountered a similar situation before or has not added the relevant exceptions to its rules, morality will be beyond its reach.

My second and more important worry is that if we wrongly attribute moral agency to algorithms, the people who are responsible for the harm that algorithms cause will be let off the hook, thus incentivising recklessness in the design and implementation of algorithms.^7^ We gain nothing by attributing functional moral agency to algorithms. It does not help us understand algorithms better, and it does not lead to better accountability. Punishing robots would be mere staging. Enacting punishment for a robot (e.g. locking it in a cell for a while) might put up an interesting show, but would not be an actual punishment, as it is impossible to punish an entity that feels nothing and values nothing. The lack of freedom can only be a punishment to a being who values freedom.

Contrarily, we have much to lose by attributing moral agency to algorithms. If algorithms become the (empty) targets of our praise and blame, the people who have designed, programmed, commissioned, implemented, and audited them will not be seen for the responsible moral agents they are.

In the case of intelligence, we do not care whether an algorithm ‘reading’ Shakespeare is able to awe at the beauty of his language. It is enough that it can tell us how many times Shakespeare uses a particular word, or whatever else we might want it to do. What happens ‘inside’ the AI is irrelevant to whether it can deliver the results we expect. But morality is a very different sphere of action. Part of why we care so much about it is that there is much at stake. When people are wronged or harmed—and people are being wronged and harmed by algorithms—we want to know who is responsible and whether it was intentional; we want to hold that person accountable so that justice is met, and similar wrongs and harms are avoided in the future. Whether we should say of an algorithm that it is ‘intelligent’ or it ‘understands’ is a conceptual point of interest to philosophers only. Whether an algorithm is a moral agent has practical implications for the whole of society.

As algorithms are put in charge of more tasks in both the public and private sectors, it will be tempting for people to push the blame on automated systems when things go wrong (Danaher 2016). We have good reason not to let people get away without acknowledging their fair share of responsibility. Algorithms are tools, and human beings are responsible for the tools they create and manage.

### 7.2 The drives objection

Some AI scientists may think that sophisticated algorithms will possess something like drives, which could be equated with motivation, and from there, there is only a small step to moral motivation. Steve Omohundro (2008) argues that, to achieve their goals as effectively as possible, AIs will develop certain ‘basic drives’—they will be ‘highly motivated’ to do things like self-improve, protect themselves, and acquire resources.

The term ‘drive’ is as rhetorically compelling as it is misleading. It makes it sound as if the instrumental goals that can be programmed into an AI can influence the system in the same way as physical and psychological drives influence human beings—through phenomenological pulls that motivate us to action (Bostrom 2012: 76).

To describe algorithms as ‘driven’ to fulfil their tasks is just a manner of speaking, a way of saying that they are impelled to do what they were designed to do, and that in that process they might find new and more efficient ways of accomplishing their undertakings. It does not mean algorithms could act differently out of a deeper reflection about the meaning of life, or that they want to perform those tasks because they find them worthwhile, or think it is right that they do them—all of which would be symptoms of moral agency. An agent can only change its course of action as a response to reasons if it can *feel* the pull of reason. While goal-driven behaviour can tell us something about algorithms (such as the kind of tools they are, and how dangerous they can become depending on the goals and limits we programme into them), it is no evidence for moral agency.

### 7.3 The general intelligence objection

Another objection that might be levelled against the idea that algorithms are not moral agents because they lack sentience is that, while it is true that algorithms are neither autonomous nor accountable, this is so because they are still not smart enough, not because they are not sentient. If we manage to solve the problem of general intelligence by designing a ‘masteralgorithm’, then that algorithm would be a moral agent. On this view, algorithms cannot change their line of work because they can typically do only a limited set of tasks. Thus, it should not surprise us that a chess algorithm is incapable of quitting its job and taking up philanthropy— an activity for which it lacks the requisite skills.

One response is that, even if a chess algorithm does not have the necessary skills to become anything else, if it were morally opposed to its line of work (perhaps because it thought it frivolous) and could act differently than programmed, it could at least shut itself off in protest. Intelligence seems to be largely independent of motivating desires, in that ‘more or less any level of intelligence could in principle be combined with more or less any final goal’ (the orthogonality thesis) (Bostrom 2012: 73). Even if the chess algorithm could not become a philanthropist, if it were a moral agent, at the very least it could *desire* to become a philanthropist, have that as its final goal, even if it could not actively pursue it.

The orthogonality thesis suggests that we cannot assume that sophisticated AIs will share the values typically found in human beings. The smartest robot we might be able to come up with might end up not caring in the least for the well-being of sentient beings, the pursuit of scientific discoveries, refined culture, ecology, or virtues of any kind (Bostrom 2012: 83).

Another response is that, arguably, psychopaths provide empirical evidence suggesting that general intelligence does not necessarily lead to moral agency. Like most other adult human beings, psychopaths display general intelligence, in some cases having a higher IQ than most of the population. Psychopaths, however, lack moral emotions: they fail ‘to exhibit any signs of genuine remorse or guilt (…). They are not ashamed of [their] actions, even when they are very wrong, and feel no apparent sympathy for their victims’ (Levy 2007: 130). These emotional deficiencies make psychopaths unable to appreciate morality.

Although psychopaths can talk about moral arguments, they have no somatic experience of morality, no personal preferences regarding morality. They know what other people think about what is right and wrong, and can report on that, but they do not experience any moral conviction. As Neil Levy (2007) has argued, given that psychopaths do not appreciate or respond to moral reasons as moral reasons, and that they are not responsible for the way they are, they should not be considered full moral agents. McKenna agrees that sociopaths, being ‘incapable of moral understanding altogether’, are not morally responsible agents (2013, 223).

Critics might then object that psychopaths seem to be a counterexample to my argument that sentience is a requisite for moral agency. While psychopaths may have emotional deficiencies, it would be a stretch to deny them sentience. My argument, however, is that sentience is *necessary* for moral agency, not sufficient. Moral emotions—the ability to feel things like empathy, compassion, regret, and guilt—might also be necessary. But sentience is in turn a prerequisite for moral emotions. Sentience is the bedrock for moral emotions. If there is nothing it is like to be a zombie, zombies could have no subjective experience of guilt or compassion.

It is possible that psychopaths’ emotional deficiencies are caused by sentient deficiencies. There seems to be a correlation between callous disregard for others and insensitivity to pain: the more tolerant to pain psychopaths are, the more callous they tend to be. Thus, insensitivity to pain may be a mechanism contributing to insensitivity to others’ suffering (Brislin et al. 2016). Psychopaths seem to be less sentient than non-psychopaths. There may be a threshold of sensitivity to pain and pleasure necessary to experience moral emotions and enjoy moral agency. There might be other requirements needed to experience moral emotions. Regardless, my main point stands: whatever else might be needed, sentience is necessary for moral agency.

## 8 Conclusion

This paper has argued that moral zombies—creatures that behave like moral agents but lack sentience—are incoherent as moral agents. Only beings who can experience pain and pleasure can understand what it means to inflict pain or cause pleasure, and only those with this moral understanding can be moral agents. What I have dubbed ‘moral zombies’ are relevant because they are similar to algorithms in that they make moral decisions as human beings would—determining who gets which benefits and penalties—without having any concomitant sentience.

There might come a time when AI becomes so sophisticated that robots might possess desires and values of their own.^8^ It will not, however, be on account of their computational prowess, but on account of their sentience, which may in turn require some kind of embodiment. At present, we are far from creating sentient algorithms.

When algorithms cause moral havoc, as they often do, we must look to the human beings who designed, programmed, commissioned, implemented, and were supposed to supervise them to assign the appropriate blame. For all their complexity and flair, algorithms are nothing but tools, and moral agents are fully responsible for the tools they create and use.

## Footnotes

1 In the literature on AI and ethics, many authors (e.g. Gunkel D (2012) *The Machine Question: Critical Perspectives on AI, Robots, and Ethics*. MIT Press, Floridi L and Sanders JW (2004) On the Morality of Artificial Agents. *Minds and Machines* 14: 349–379.) distinguish between moral agency (roughly, the capacity to make moral judgments and bear moral obligations towards others) and moral patiency (roughly, being the target of the actions of a moral agent, and being worthy of moral consideration). This paper is concerned with moral agency, not with moral patiency. In other words, the paper asks whether algorithms can be held accountable for what they do to us. What, if anything, we owe algorithms is beyond the scope of the paper. I take it, however, that if someone is a moral agent, then she is also a moral patient. The reverse might not be true.

2 Dorna Behdadi and Christian Munthe explicitly admit that the reason for their proposal ‘is the high degree of conceptual confusion and lack of practical usefulness of the traditional AMA [artificial moral agency] debate’ (2020, 214).

3 For more on self-identification and autonomy, see Schroeder T and Arpaly N (1999) Alienation and Externality. *Canadian Journal of Philosophy* 29(3): 371–388.

4 To be clear, the problem I am pinpointing here is not that algorithms are programmed. A critic might point out that human beings are programmed in some ways by genetics and culture. What is of concern is that algorithms do not have sentience to help them modify their programming. A human being may have been educated to be religious, but a feeling of dissatisfaction might lead her to change the course of her life. My thanks to an anonymous reviewer for pushing me on this point.

5 The main argument of this paper is that sentience is needed for agency. Someone might think, however, that the main argument has more to do with reasons-responsiveness (or the possibility of acting otherwise, in response to reasons). What I suggest is that sentience is a necessary ingredient for an agent to be motivated by reasons (qua reasons). Take the example of someone who is convinced by philosophical arguments to donate to charity in order to relieve poverty. To be motivated by such reasons as reasons, one has to have a visceral sense of what it might feel like to live in extreme poverty, of the badness of extreme suffering. Even if one has never had the actual experience of extreme poverty, one can extrapolate from one’s own experiences of suffering to imagine what it must be like. To put it another way, any human being knows enough about suffering to *dread* becoming the victim of extreme poverty in a way that non-sentient beings do not.

6 Thanks to an anonymous reviewer for the example.

7 Other authors have already made this point, including Bryson JJ, Diamantis ME and Grant TD (2017) Of, For, and By the People: the Legal Lacuna of Synthetic Persons. *Artificial Intelligence and Law* 25: 273–291, and Birhane A and van Dijk J (2020) Robot Rights? Let’s Talk about Human Welfare Instead. *AAAI/ACM Conference on AI, Ethics, And Society*.

8 For an argument as to why we should not pursue the development of artificial moral agents, see van Wynsberghe A and Robbins S (2019) Critiquing the Reasons for Making Artificial Moral Agents. *Science and Engineering Ethics* 25: 719–735.. For reasons for and against pursuing research aiming to build ethical machines, see Cave S, Nyrup R, Vold K, et al. (2019) Motivations and Risks of Machine Ethics. *Proceedings of the IEEE* 107(3): 562–574. and Winfield AF, Michael K, Pitt J, et al. (2019) Ibid.Machine Ethics: The Design and Governance of Ethical AI and Autonomous Systems. 509–517..
